# A Novel Non-Isolated High-Gain Non-Inverting Interleaved DC–DC Converter

**DOI:** 10.3390/mi14030585

**Published:** 2023-02-28

**Authors:** Farhan Mumtaz, Nor Zaihar Yahaya, Sheikh Tanzim Meraj, Narinderjit Singh Sawaran Singh, Ghulam E Mustafa Abro

**Affiliations:** 1 Department of Electrical and Electronics Engineering, Universiti Teknologi PETRONAS, Seri Iskandar 32610, Perak, Malaysia; 2 Faculty of Data Science and Information Technology, INTI International University, Persiaran Perdana BBN Putra Nilai, Nilai 71800, Negeri Sembilan, Malaysia

**Keywords:** non-isolated DC–DC converter, non-inverting, high gain, switching stress, renewable energy

## Abstract

High-gain DC–DC converters are being drastically utilized in renewable energy generation systems, such as photovoltaic (PV) and fuel cells (FC). Renewable energy sources (RES) persist with low-level output voltage; therefore, high-gain DC–DC converters are essentially integrated with RES for satisfactory performance. This paper proposes a non-isolated high-gain non-inverting interleaved DC–DC boost converter. The proposed DC–DC converter topology is comprised of two inductors and these are charging and discharging in series and parallel circuit configurations. The voltage multiplier technique is being utilized to produce high gain. The proposed topology is designed to operate in three modes of operation. Three switches are operated utilizing two distinct duty ratios to avoid the extreme duty ratio while having high voltage gain. Owing to its intelligent design, the voltage stress on the switches is also significantly reduced where the maximum stress is only 50% of the output voltage. The proposed converter’s steady-state analysis with two distinct duty ratios is thoroughly explored. Furthermore, a 160 W 20/400 V prototype is developed for performance analysis and validation. The converter topology can generate output voltage with a very high voltage gain of 20, which is verified by the prototype. Moreover, a high efficiency of 93.2% is attained by the proposed converter’s hardware prototype.

## 1. Introduction

In recent years, energy generation using renewable energy sources (RES) has significantly contributed to sustainable development globally [1,2]. Photovoltaic (PV) has gained remarkable popularity among other renewable energy sources. PV systems have already outperformed with both on-grid and off-grid-connected systems [3,4]. Power generation systems utilizing RES generates a low-level output voltage; therefore, requisite effective DC–DC converters having higher voltage gain capability [5]. Other than renewable power conversion, numerous other applications utilize DC–DC converters, such as electric vehicles, electric traction systems, power back-up systems, surgical equipment, and lighting applications [6,7]. In earlier times, conventional DC–DC converters were opted for voltage-boosting applications. However, conventional DC–DC boost converters persist with high switching stress that is equivalent to the output voltage [8]. Therefore, it requires switches with higher power ratings that ultimately increase the conduction losses to cater to the higher switching stress. Furthermore, choosing higher duty ratios to acquire high voltage gain induces high voltage spikes, conduction losses, and generates diode reverse recovery issues [9].

Nowadays, various DC–DC converter topologies with high-gain capabilities are available as per the application requirements [10]. High-gain DC–DC converters are further distributed within two clusters, named as isolated and non-isolated converter topologies [11]. Numerous isolated converter topologies to acquire high voltage gain are elaborated in the literature [12]. However, isolated converter topologies have substantial problems that include thermal impact, high voltage ripples on the power switches, leakage inductance, core saturation, and their large size makes them more expensive [11,13]. Thus, non-isolated converter topologies are opted for instead, for higher voltage gain; these are smaller in size and cost-effective, considering that no galvanic isolation is required [14,15].

Non-isolated converter topologies are also designed for specific applications, such as electric vehicles (EV). In ref. [16], three-phase interleaved parallel bi-directional converter topology is integrated with EV that offer multi-phase circuit topology, and the operation is based on multiple stages with different duty cycle ranges in order to fulfil the EV power requirements in a smooth manner. Another multi-phase interleaved boost converter topology using an auxiliary resonant circuit is presented in ref. [17]; it has the capability of soft switching and high voltage gain. For fuel cell integration non-isolated interleaved high-gain converter topologies are presented in ref. [18,19]; these interleaved topologies offer an improved DC bus regulation for the fuel cell irrespective of the intermittent source voltage. Furthermore, within the category of non-isolated high-gain converters most widely utilized, the topologies are quadratic boost [20], with a wide range of duty cycle ratios usually operated with a single switch; however, it has higher switching and diode stress in comparison to other non-isolated converter topologies. In ref. [21], a cascaded boost converter utilizes the initial stage for voltage boosting with a higher duty ratio, whereas the second stage operates with a nominal duty ratio with lower switching stress in comparison to the first stage of the converter topology. Besides high-gain capability, it persists with some severe issues that include higher component count that results in poor efficiency, and it also possesses a diode reverse-recovery issue. Coupled inductor cascaded boost is presented in ref. [22], and significantly high voltage gain could be attained by utilizing an extreme duty ratio for the operation of the converter or through increasing the coupled inductor’s turns ratio; it is considered a design tradeoff. Due to excessive current stress across the switch, it is incompatible with the high-power applications. Another converter topology utilizing the voltage lifting approach is demonstrated in ref. [23]; it overcomes the effect of parasitic elements in DC–DC converters and has improved power transfer efficiency. However, it has high switching stress and high passive component count, and is, therefore, not suitable for high-power applications. In ref. [24], an active–passive inductor cell (APIC) converter topology is presented. Higher voltage gain along with optimal duty cycle are the highlighted merits for the proposed topology. In contrast, the high-power application requires higher active-passive inductor cells that will increase the complexity of the converter and will have high stress on the switches. However, with the addition of a switched-capacitor or switched-capacitor inductor, a power converter’s complexity, along with the cost, increases significantly.

Various coupled inductor integrated DC–DC converter topologies are capable of generating higher gain factor along with less or optimum switching stress switches relying on the duty cycle ratio variation [25]. In certain cases, to attain the requisite voltage conversion set-point, the inductor turns ratio is increased; this results in an excessive input current ripple [26]. Therefore, the input current ripple requires optimization utilizing the filter at the input side, as depicted in ref. [27]. In ref. [28], a single-switch and single-inductor cascaded converter topology is presented, to acquire higher gain factor along with less input current ripple; however, it has quite a high component count and also possess harmonics due to capacitors; hence, it is not advisable for high-power loads. Furthermore, high-gain factor hybrid converter topologies are discussed in refs. [24,29,30].

This paper presents, a high-gain non-inverting interleaved boost converter topology to resolve the aforementioned issues. By using appropriate component values and suitable duty ratios, the proposed topology produces higher voltage gain. Furthermore, the proposed topology has these subsequent advantages:

(1) The proposed converter topology is operated utilizing two distinct duty cycle ratios to achieve higher voltage gain.
(2) The energy stored in the inductor is delivered to the voltage multiplier and supplied to the load.
(3) The proposed converter topology achieves significantly higher voltage gain factor in comparison to the conventional boost and other high-gain converter topologies proposed in refs. [8,22,23,30,31,32,33,34].
(4) Voltage stress is significantly lower across the diodes and the switches as compared to the voltage output percentage.

Section 2 discusses the circuit arrangement of the proposed boost converter topology in detail. Steady-state analysis for the proposed DC–DC boost converter topology is illustrated in Section 3. Section 4 elaborates on the efficiency evaluation of the proposed topology. A performance evaluation of the proposed topology with respect to the voltage stress across the diodes, switches, and voltage gain is discussed in Section 5. Hardware results of the proposed converter topology is described in Section 6. Furthermore, a conclusion of the results for the proposed topology is highlighted in Section 7.

## 2. Circuit Configuration

**Figure 1:**
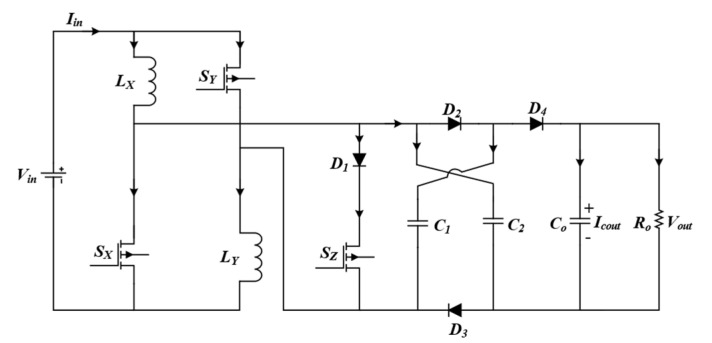
Proposed non-isolated high-gain non-inverting interleaved converter.

The circuit of the proposed converter topology is demonstrated in Figure 1, which is comprised of three power switches, *S*_X_, *S*_Y_, and *S*_Z_, two inductors, *L*_X_ and *L*_Y_, four diodes, *D*_1_, *D*_2_, *D*_3_, and *D*_4_, and three capacitors, *C*_1_, *C*_2_, *C*_o_. The power switches, *S*_X_, *S*_Y_, and *S*_Z_, are operated with a switching frequency *f*_sw_. The switches, *S*_X_ and *S*_Y_, are operated with a duty ratio denoted as *D*_1_, whereas *S*_Z_ is operated with a duty ratio *D*_2_.

Some suppositions are considered while explaining the proposed converter topology’s steady-state operation, including: (1) All of the components in the circuit are ideal. The impact of forward voltage drop, switch ON state resistance, and equivalent series resistance (ESR) for the capacitors and the inductors are ignored; (2) To maintain the stable output voltage, the output capacitor *C*_o_ is adequately large. Assume that the two inductors have an equivalent number of turn ratios.

(1) $$L_{X}=L_{Y}=L$$

Therefore, a similar voltage is across both the inductors, *V_LX_* and *V_LY_*, depicted in Equations (2) and (3).

(2) $$V_{\mathrm{LX}}=L_{X}\frac{diL_{X}}{dt}=L\frac{diL_{X}}{dt}$$

(3) $$V_{\mathrm{LY}}=L_{Y}\frac{diL_{Y}}{dt}=L\frac{diL_{Y}}{dt}$$

## 3. Proposed Converter’s Steady-State Analysis

**Figure 2:**
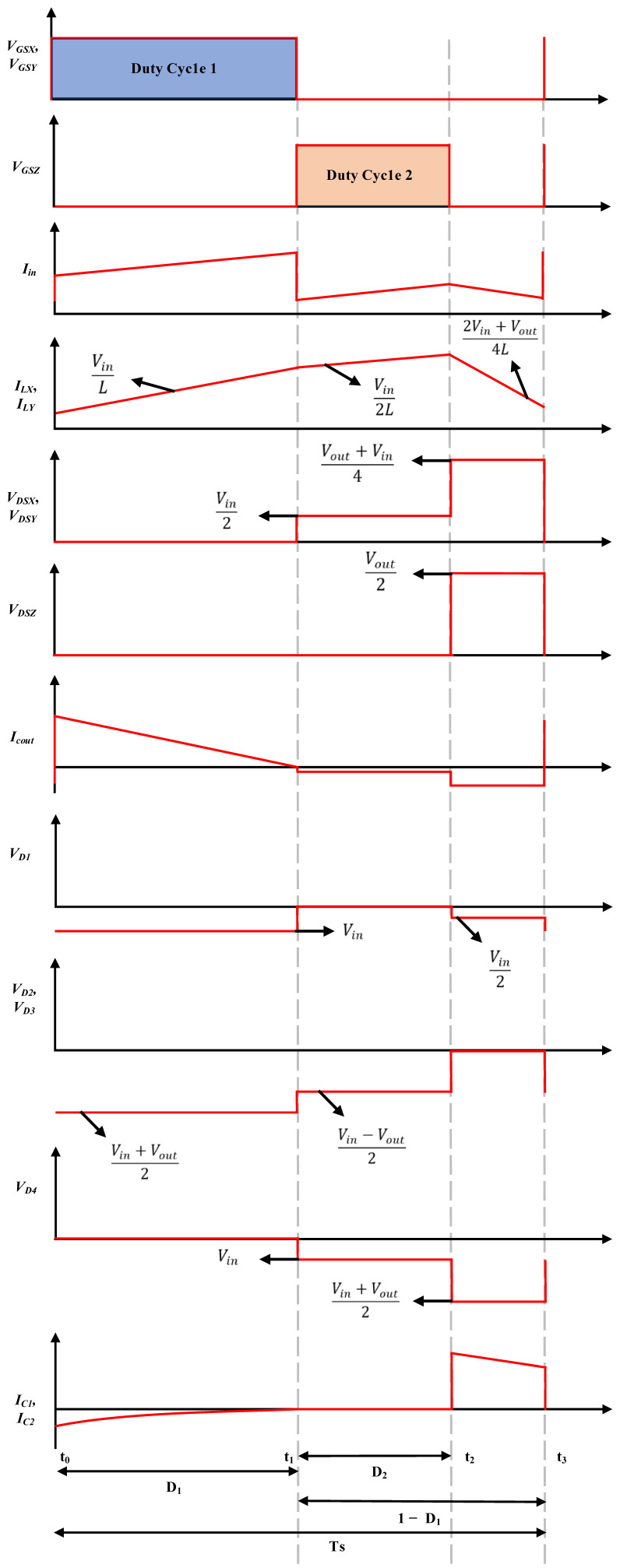
CCM operation of the proposed DC–DC boost converter topology.

This section elaborates on the operating modes in continuous conduction mode (CCM) of the proposed boost converter topology. There are three operational modes with two distinct duty ratios within a single switching period for the proposed converter. The output response in CCM for the proposed topology is illustrated in Figure 2.

### 3.1. Working in Continuous Conduction Mode

**Figure 3:**
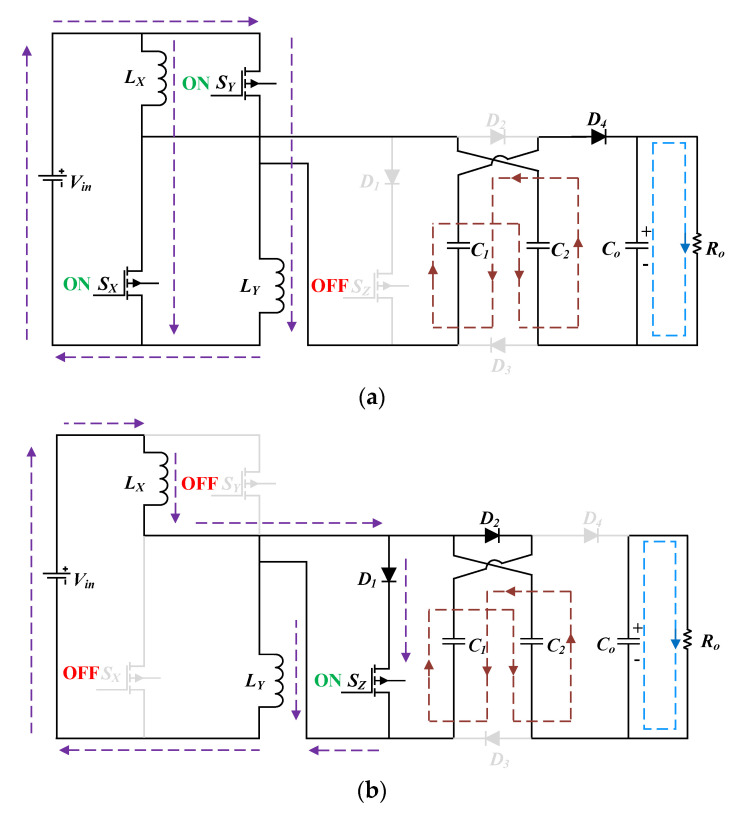

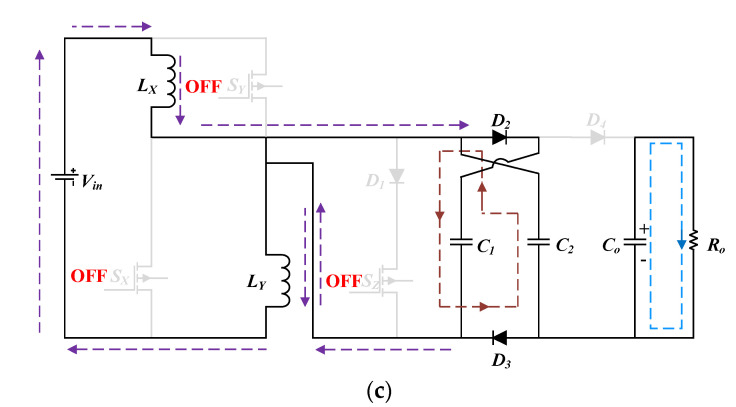
Operational modes in CCM: (**a**) mode I; (**b**) mode II; (**c**) mode III.

Mode I: The mode I time interval is [*t*_o_ − *t*_1_]; switches *S*_X_ and *S*_Y_ are turned ON, whereas the third switch *S*_Z_ remains in the OFF state. Throughout this operation, the circuit’s current direction is illustrated in Figure 3a. Here, the input energy will be supplied to the two inductors *L*_X_ and *L*_Y_, and the capacitor *C*_o_ will supply stored energy at the output. The diodes *D*_1_ and *D*_4_ remains in reversed bias, whereas the inner diode of the third switch *S*_Z_ remains in the forward-biased state. Thus, even if the third switch *S*_Z_ remains in an OFF state, the conduction voltage will be present across it. As the inductors and the source are parallel to each other in this mode, the inductor voltages are presented as follows:

(4) $$V_{\mathrm{LX}}=V_{\mathrm{LY}}=V_{in}$$

To obtain Equations (5), (2) and (3) are substituted into Equation (4)

(5) $$L\frac{diL_{X}}{dt}=L\frac{diL_{Y}}{dt}=L\frac{diL}{dt}=V_{in},\;\;\;\;t_{o}\leq t\leq t_{1}$$

(6) $$\frac{diL_{X}}{dt}=\frac{diL_{Y}}{dt}=\frac{diL}{dt}=\frac{V_{in}}{L}$$

(7) $$\frac{diL}{dt}=\frac{V_{in}}{L}$$

Mode II: The mode II time interval is [*t*_1_ − *t*_2_]; the third switch *S*_Z_ is turned ON, whereas the two switches *S*_X_ and *S*_Y_ remains in the OFF state. Thus, the current direction of the circuit in mode II is demonstrated in Figure 3b. The two inductors *L*_X_, and *L*_Y_ are fed by the input source, and the circuit current flows through *L*_X_, *D*_1_, *D*_4_, and *L*_Y_. The switching stress on *S*_X_ and *S*_Y_ in this mode is half of the supplied voltage. Furthermore, the output capacitor *C*_o_ delivers the accumulated energy at the output load as *D*_4_ is not in a forward-biased condition. The two inductors and the input source are connected in this mode. Expressions for calculating the inductor current and voltage are as follows:

(8) $$iL_{X}=iL_{Y}=iL$$

(9) $$V_{\mathrm{LX}}+V_{\mathrm{LY}}=V_{in}$$

(10) $$L\frac{diL_{X}}{dt}+L\frac{diL_{Y}}{dt}=V_{in}$$

Since the two inductors *L*_X_ and *L*_Y_ are coupled in series to the source voltage *V_in_*, and *i*_L_ current flowing through the inductors *L*_X_ and *L*_Y_. By substitution of (2) and (3) into (8), the subsequent expression is attained:

(11) $$\frac{diL}{dt}=\frac{V_{in}}{2L},\;\;\;\;t_{1}\leq t\leq t_{2}$$

Mode III: The mode III time interval is [*t*_2_ − *t*_3_]; and all three switches *S*_X_, *S*_Y_, and *S*_Z_ are in the OFF state. Figure 3c depicts the circuit’s current path in mode III. Thus, in mode III, the input source together with the both inductors feed the output load. The diode *D*_1_ is under the non-conduction state, being reverse-biased. Additionally, *D*_2_ is in the forward-biased state that enables output capacitor *C*_o_ to be charged in this mode. The switching stress across *S*_X_ and *S*_Y_ is half of the average of source voltage and the output voltage, while for the voltage stress, the third switch *S*_Z_ is half related to the output voltage.

The two inductors *L*_X_ and *L*_Y_ are coupled in series to the input source in this mode. Expressions for calculating the inductor current and voltage are as follows:

(12) $$iL_{X}=iL_{Y}=iL$$

(13) $$VL_{X}+VL_{Y}=V_{in}-\frac{V_{out}}{2}$$

(14) $$2L\frac{diL}{dt}=V_{in}-\frac{V_{out}}{2}$$

where the converter’s output voltage is *V_out_*. By substitution of Equations (2) and (3) into Equation (13) and simplifying Equation (14), the following expression (15) is attained: voltage. Both the inductors are coupled in a series connection to the input source in this mode. Expressions for calculating the inductor’s current and voltage is attained as follows:

(15) $$\frac{diL}{dt}=\frac{2V_{in}-V_{out}}{4L},\;\;\;\;t_{2}\leq t\leq t_{3}$$

By implying the state-space averaging technique, the subsequent expressions are attained from Equations (7), (11) and (15):

(16) $$\int_{0}^{D_{1}T_{s}}{\left(\frac{diL}{dt}\right)}^{I}dt+\int_{0}^{D_{2}T_{s}}{\left(\frac{diL}{dt}\right)}^{II}dt+\int_{0}^{(1-D_{1}-D_{2})T_{s}}{\left(\frac{diL}{dt}\right)}^{III}dt=0$$

Simplifying Equation (16), the voltage gain expression is obtained:

(17) $$\frac{V_{out}}{V_{in}}=\frac{2\left(D_{1}+1\right)}{\left(1-D_{1}-D_{2}\right)}$$

### 3.2. Switching Stress

The switching stress on all three switches *V_DSX_*, *V_DSY_*, and *V_DSZ_* is depicted in Figure 2, correspondingly, and is expressed as follows:

(18) $$\left\{\begin{array}{c} V_{DS1}=V_{DS2}=\frac{V_{in}+V_{out}}{4}\\ V_{DS3}=\frac{V_{out}}{2} \end{array}\right.$$

### 3.3. Diode Voltage Stress

The diode voltage stress *V_D_*_1_, *V_D_*_2_, *V_D_*_3_, and *V_D_*_4_ on the diodes *D*_1_, *D*_2_, *D*_3_, and *D*_4_ is expressed as follows:

(19) $$\left\{\begin{array}{c} V_{D1}=V_{in}\\ V_{D2}=V_{D3}=V_{D4}=\frac{V_{in}+V_{out}}{2} \end{array}\right\}$$

### 3.4. Component Selection

To obtain the appropriate performance of converter topology, adequate component selection is critical. The proposed converter topology’s component selection comprises suitable inductor and capacitor calculations.

#### 3.4.1. For Inductor

The appropriate inductor selection [35] relies on the input voltage *V_in_*, duty ratio *D*_1_, ripple current Δ*i_L_*, and the switching frequency *f_sw_*. For the optimum operation of proposed converter topology in CCM mode, an appropriate inductor value is determined by the following equation:

(20) $$L_{X}=L_{Y}=\frac{V_{in}\times D_{1}}{\Delta iL\times f_{sw}}$$

#### 3.4.2. For Capacitor

Obtain optimum values for the selection of the output capacitor *C*_o_ relies on the output voltage *V_out_*, ripple voltage Δ*V_c_*, switching frequency *f_sw_*, and the output power of the converter *P_out_*. These are attained by using the following expression:

(21) $$C_{out}=\frac{P_{out}}{V_{out}\times \Delta V_{c}\times f_{sw}}$$

Respectively, the capacitance value for *C*_1_ and *C*_2_ are similar in order to avoid voltage distortions.

## 4. Efficiency Evaluation

**Figure 4:**
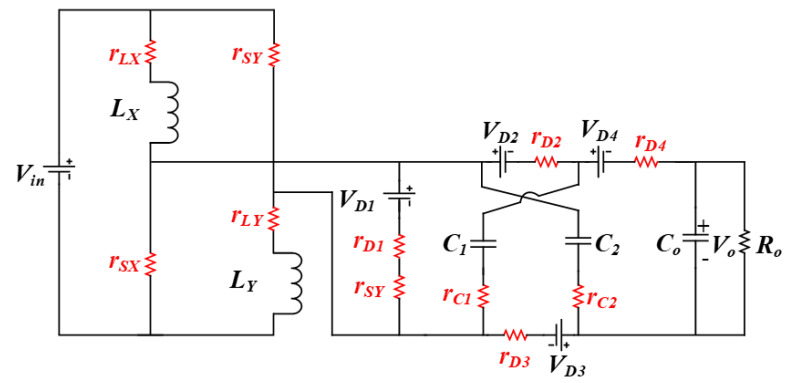
Proposed converter’s equivalent circuit.

The efficiency evaluation of the proposed converter topology is discussed with details for each of the operational modes. Figure 4. depicts the proposed converter’s equivalent circuit. Thus, equivalent series resistance (ESR) for both of the inductors *L*_X_ and *L*_Y_ are denoted as *r_L__X_* and *r_L__Y_*. Furthermore, ESR for the two capacitors *C*_1_ and *C*_2_ are denoted by *r_C_*_1_, *r_C_*_2_. Correspondingly, for the diodes *D*_1_, *D*_2_, *D*_3_, and *D*_4_, their internal resistances are represented by *r_D_*_1_, *r_D_*_2_, *r_D_*_3_ and, *r_D_*_4_ and the voltage drop on the diodes are denoted by *V_D_*_1_, *V_D_*_2_, *V_D_*_3_ and *V_D_*_4_. Similarly, ON-state resistance for all the three switches *S*_X_, *S*_Y_ and *S*_Z_ are indicated by *r_S_*_X_, *r_S_*_Y_, and, *r_S_*_Z_.

*Mode I:* The time interval for mode I is *D*_1_*Ts*; the two switches *S*_X_ and *S*_Y_ are in an ON state while the third switch *S*_Z_ remains in an OFF state; the average capacitor current *I_cout_* and the inductor voltage *V_LX_* are expressed as:

(22) $$I_{cout}^{I}=i_{LX}-\frac{V_{out}}{R_{o}}$$

(23) $$V_{LX}^{I}=V_{in}-i_{LX}\left(r_{LX}+r_{SX}\right)$$

*Mode II:* The time interval for mode II is *D*_2_*Ts*; the two switches *S*_X_ and *S*_Y_ are in an OFF state, whereas the third switch *S*_Z_ is in an ON state; the average capacitor current *I_cout_* and the inductor voltage *V_LX_* are expressed as:

(24) $$I_{cout}^{II}=-\frac{V_{out}}{R_{o}}$$

(25) $$V_{LX}^{II}=\frac{V_{in}-I_{LX}\left(r_{LX}+r_{LY}\right)-I_{in}\left(r_{D2}+r_{SZ}\right)-V_{D1}}{2}$$

*Mode III:* The time interval for mode III is $(1-D_{1}-$ *D*_2_); all the switches *S*_X_, *S*_Y_, and *S*_Z_ are in an OFF state, while the average capacitor current *I_cout_* and the inductor voltage *V_LX_* are expressed as:

(26) $$I_{cout}^{III}=-\frac{V_{out}}{R_{o}}$$

(27) $$V_{LX}^{III}=\frac{V_{in}-I_{LX}\left(r_{LX}+r_{LY}\right)-\frac{V_{out}}{2}-\frac{V_{out}}{2}\left(r_{C2}+r_{D3}\right)-V_{D3}}{2}$$

To obtain Equation (28), an ampere-second balance approach is implemented for the output capacitor *C*_o_.

(28) $$\int_{0}^{D_{1}T_{s}}I_{co}^{I}dt+\int_{0}^{D_{2}T_{s}}I_{co}^{II}dt+\int_{0}^{(1-D_{1}-D_{2})T_{s}}I_{co}^{III}dt=0$$

Inductor current through *I_LX_* is obtained by simplifying Equation (28):

(29) $$I_{LX}=-\frac{V_{out}}{R_{o}D_{1}}$$

By implementing a volt-second balance approach to the inductor *L*_X_, expression (30) is attained.

(30) $$\int_{0}^{D_{1}T_{s}}V_{LX}^{I}dt+\int_{0}^{D_{2}T_{s}}V_{LX}^{II}dt+\int_{0}^{(1-D_{1}-D_{2})T_{s}}V_{LX}^{III}dt=0$$

The output voltage obtained in (31) is achieved by simplifying (30).

(31) $$V_{out}=\frac{2[V_{in}\left(1+D_{1}\right)-(V_{D1}D_{2})-(V_{D3}\left(1-D_{1}-D_{2}\right))]}{\left(\frac{D_{1}X_{1}+D_{2}X_{2}+X_{3}}{R_{o}D_{1}}\right)+\left(1-D_{1}-D_{2}\right)}$$

where,

$$X_{1}=2r_{LX}+4r_{SX}-r_{C2}-r_{D3}-r_{D4}-2r_{LY}$$

(32) $$X_{2}=\frac{2\left(r_{D2}-r_{SZ}\right)\left(r_{D2}-r_{C1}\right)\left(r_{D3}-r_{C2}\right)}{\left(r_{D1}-r_{SZ}\right)\left(r_{D2}+r_{C1}+r_{D3}+r_{C2}\right)+\left(r_{D2}-r_{C1}\right)\left(r_{D3}-r_{C2}\right)}-2r_{C2}-2r_{D3}-2r_{D4}$$

$$\;X_{3}=2r_{LX}+2r_{LY}+r_{C2}+r_{D3}+r_{D4}$$

The expressions for the input and output powers are as follows:

(33) $$P_{in}=2V_{in}I_{LX}D_{1}+V_{in}I_{LX}D_{2}+V_{in}I_{LX}\left(1-D_{1}-D_{2}\right)$$

The input power is attained by substituting (29) into (33).

(34) $$P_{in}=\frac{V_{in}V_{out}\left(1+D_{1}\right)}{R_{o}\left(1-D_{1}-D_{2}\right)}$$

(35) $$P_{out}=\frac{V_{out}^{2}}{R_{o}}$$

The efficiency evaluation of the proposed converter topology related to conduction losses is expressed in Equation (37), and calculated by using Equations (31), (34) and (35).

(36) $$\eta =\frac{P_{out}}{P_{in}}$$

(37) $$\eta =\frac{2\left[V_{in}\left(1+D_{1}\right)-V_{D1}D_{2}-V_{D3}\left(1-D_{1}-D_{2}\right)\right]D_{1}}{V_{in}\left(1+D_{1}\right)\left(1-D_{1}-D_{2}\right)+\left[\frac{\left(D_{1}X_{1}+D_{2}X_{2}+X_{3}\right)}{R_{o}D_{1}}\right]}$$

Considering the switching losses (*P_sw_*), expression (38) is derived for the output power for the proposed converter topology.

(38) $$P_{out}=\frac{V_{out}^{2}}{R_{o}}-P_{sw}$$

(39) $$P_{sw}=V_{DS}I_{D}\left(t_{rise}+t_{fall}\right)f_{sw}$$

Thus, *V_DS_* is the MOSFET’s voltage from drain to source, *I_D_* represents the drain current of the MOSFET, *f_sw_* represents switching frequency, and *t_rise_* and *t_fall_* are the MOSFET’s rise time and fall time. The proposed converter’s efficiency is expressed in Equation (40), which is calculated by using Equations (31), (32), (34) and (39).

(40) $$\eta =\frac{\left[\frac{V_{out}^{2}}{R_{o}}-P_{sw}\right]}{\left[\frac{V_{in}V_{out}\left(1+D_{1}\right)}{R_{o}D_{1}}\right]}$$

Considering the capacitor losses for the proposed converter, the output power is attained as:

(41) $$P_{out}=\frac{V_{out}^{2}}{R_{o}}-P_{rc}$$

(42) $$P_{rc1}={\left\{\frac{D_{1}+\left(A-1\right)D_{2}\left(1+D_{1}\right)}{\left(1-D_{1}-D_{2}\right)}\right\}}^{2}\frac{P_{o}}{R}r_{C}$$

(43) $$P_{rc2}={\left\{\frac{D_{1}+\left(A-1\right)D_{2}\left(1+D_{1}\right)}{\left(1-D_{1}-D_{2}\right)}\right\}}^{2}\frac{P_{o}}{R}r_{C}$$

(44) $$P_{rco}={\left\{\frac{D_{1}\left(1+3D_{1}+D_{2}\right)}{\left(1-D_{1}-D_{2}\right)}\right\}}^{2}\frac{P_{o}}{R}r_{C}$$

(45) $$A=\frac{2\left(r_{D}+r_{S3}\right)}{2r_{D}+2r_{S3}+r_{D}+r_{C}}$$

(46) $$P_{rc}=P_{rc1}+P_{rc2}+P_{rco}$$

(47) $$P_{rc}=\frac{2{\left\{D_{1}+\left(A-1\right)D_{2}\left(1+D_{1}\right)\right\}}^{2}+{\left\{D_{1}\left(1+3D_{1}+D_{2}\right)\right\}}^{2}}{{\left(1-D_{1}-D_{2}\right)}^{2}}\times \frac{P_{o}}{R}$$

Power losses for all the three capacitors *C*_1_, *C*_2_, and *C*_o_ are expressed in Equations (42), (43) and (44), respectively, whereas the total capacitor power losses *P_rc_* is expressed in Equation (48). The proposed converter’s efficiency considering the capacitor losses is expressed in (47).

(48) $$\eta =\frac{\left[\frac{V_{out}^{2}}{R_{o}}-P_{rc}\right]}{\left[\frac{V_{in}V_{out}\left(1+D_{1}\right)}{R_{o}D_{1}}\right]}$$

## 5. Comparative Performance Analysis

**Figure 5:**
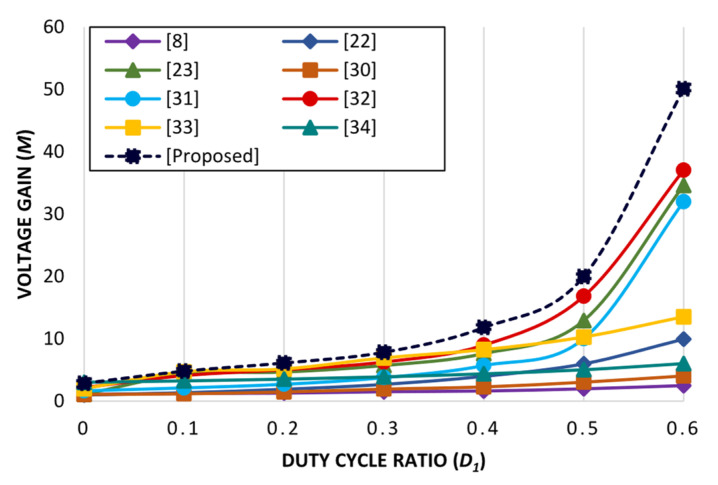
Voltage gain comparison among various recently proposed DC–DC converters.

**Figure 6:**
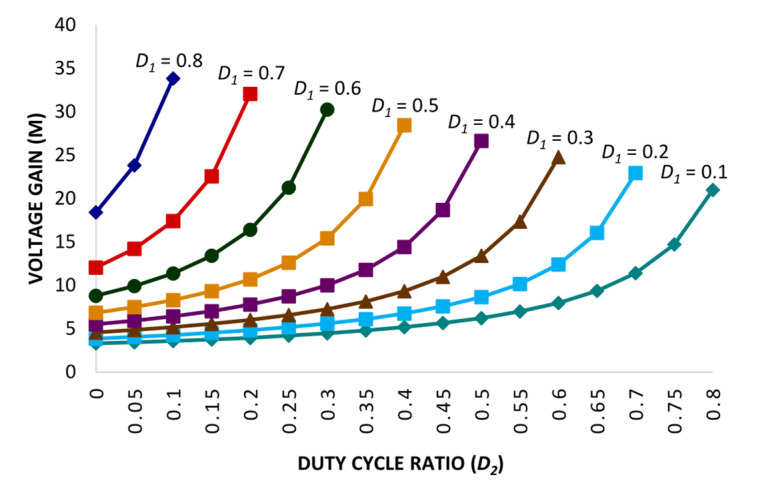
Voltage gain response under varying duty ratios *D*_1_ and *D*_2_ for the proposed converter.

The proposed converter’s characteristics comparison is determined in Table 1 with conventional and high-gain converter topologies. Comparative performance analysis of the proposed converter topology is mainly dependent on the fundamental parameters: voltage gain, switching stress, diode stress, and the quantity components. Table 1 includes converter topologies with one power switch and single duty ratio *D* presented in ref. [22] and [8] and two power switches and double duty ratios *D*_1_ and *D*_2_ [33], whereas [34] is based on two power switches and single duty ratio *D*, and three power switches and double duty ratio *D*_1_ and *D*_2_ are included in refs. [23,30,31,32]. Table 1 depicts the voltage gain equation of the proposed converter topology. It is evident that converter topologies with double duty ratios have higher gain factor and satisfactory switching stress. Similarly, the proposed topology utilizes three power switches that are operated by two distinct duty ratios, and it has a higher voltage gain, as depicted in Table 1. The calculated value of duty ratios *D*_new[p]_ is 85% where *D*_1_ = 50% and *D*_2_ = 35%. By splitting the duty ratio into two, it becomes convenient to operate the converter topology utilizing a higher duty cycle ratio within optimum operational limits; converter topologies with a single duty ratio cannot be operated by using extreme duty ratios. Moreover, the switching stress and diode stress for the proposed converter topology is less in contrast to converter topologies depicted in refs. [23,30,31,32]. Furthermore, Figure 5 depicts the assessment of the proposed converter topology and the established converter topologies with respect to voltage gain versus duty ratio. The plot depicted in Figure 5 determines the output response of the proposed topology by iterating the duty ratio *D*_1_, whereas another duty ratio *D*_2_ remains fixed at 0.35. The proposed converter has achieved a higher voltage gain factor of 50 with a duty ratio of *D*_1_ 0.6 and *D*_2_ 0.35. Whereas, converter topologies in refs. [8,22,30,34] have acquired a voltage gain of less than 15, and converter topologies in refs. [23,31,32] have obtained a voltage gain between 3 and 11. However, all the converter topologies were tested with a similar duty cycle ratio of 0.6. Moreover, Figure 6 illustrates the voltage gain response for the proposed converter topology under multiple variations in both duty ratios *D*_1_ and *D*_2_. Thus, it is shown that by applying suitable duty ratios *D*_1_ and *D*_2_, the proposed converter topology is competent for generating significant voltage gain.

**Figure 7:**
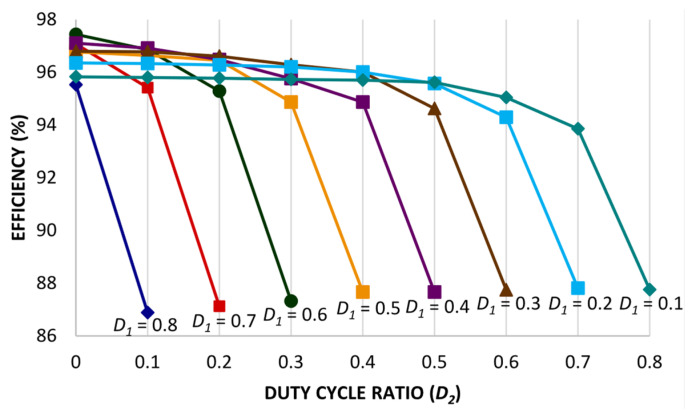
Proposed converter’s theoretical efficiency under varying duty cycle ratios *D*_1_ and *D*_2_.

Hence, the overall voltage stress for the proposed converter topology remains relatively low; for the power switches *S*_X_ and *S*_Y_, the voltage stress is 25% with respect to the output voltage, whereas the power switch *S*_Z_ has 50% voltage stress with respect to the output voltage. Moreover, the switching stress for the converters presented in ref. [8,22] is 100% of the output voltage. For converters presented in ref. [33,34], the voltage stress for switches *S*_X_ and *S*_Y_ is 50% of the output voltage. Whereas, the converter topologies presented in refs. [23,30,31,32] persist with a different switching voltage stress pattern; two of the switches *S*_X_ and *S*_Y_ have 50% voltage stress of the output voltage, and the switch *S*_Z_ has 100% voltage stress of the output voltage. However, it is evident for the proposed converter topology that it has significantly low switching voltage stress related to the converter topologies discussed in Table 1. Similarly, the voltage stress on the diodes is also low related to the converter topologies discussed in Table 1. Furthermore, the number of component counts for the converter topologies includes the quantity of switches, inductors, diodes, and capacitors. The measure of component counts for the converter topologies is between 4 and 15, since conventional and high-gain converter topologies are considered to persist with a slightly higher number of component counts. However, the component count for the proposed topology is 12, which includes three switches, three inductors, four diodes, and three capacitors. Whereas in ref. [22], the component count is 15, and the similar high-gain converter topology presented in ref. [33] has a component count of 12, equivalent to the proposed converter topology. Theoretical efficiency under varying duty ratios *D*_1_ and *D*_2_ is demonstrated in Figure 7. Thus, the efficacy of the proposed converter topology is evaluated, and remained higher than 90%. Additionally, the voltage gain for the proposed converter topology is higher than the converter topologies discussed in Table 1. Moreover, to acquire high voltage gain with optimum efficiency, the duty cycle ratio must be adequately selected considering the following limitations: (1) both the duty ratios *D*_1_ and *D*_2_ must not be equivalent to 0.5, and (2) the total duty ratios *D*_1_ and *D*_2_ must be less than 1.

## 6. Experimental Results

**Figure 8:**
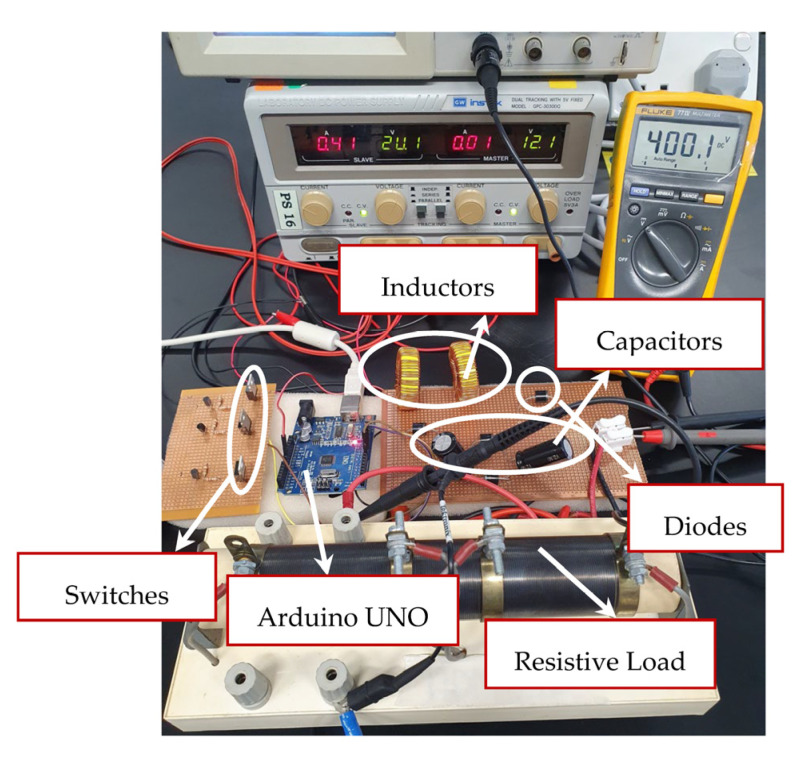
Proposed converter’s hardware prototype setup.

**Figure 9:**
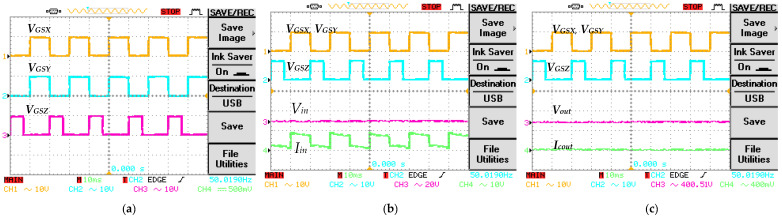

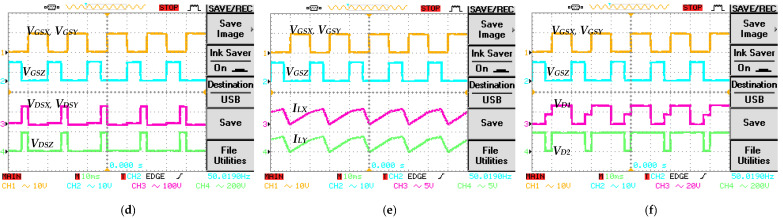
Experimental results for the proposed converter. (**a**) Gate pulse voltages (*V_GS_*_1_, *V_GS_*_2_, and *V_GS_*_3_); (**b**) input voltage and input current (*V_in_* and, *I_in_*); (**c**) output voltage and output current (*V_out_* and *i_cout_*); (**d**) switch voltages (*V_DSX_*, *V_DSY_*, and *V_DSZ_*); (**e**) inductor currents (*I_LX_* and *I_LY_*); (**f**) diode voltages (*V_D_*_1_ and *V_D_*_2_).

**Figure 10:**
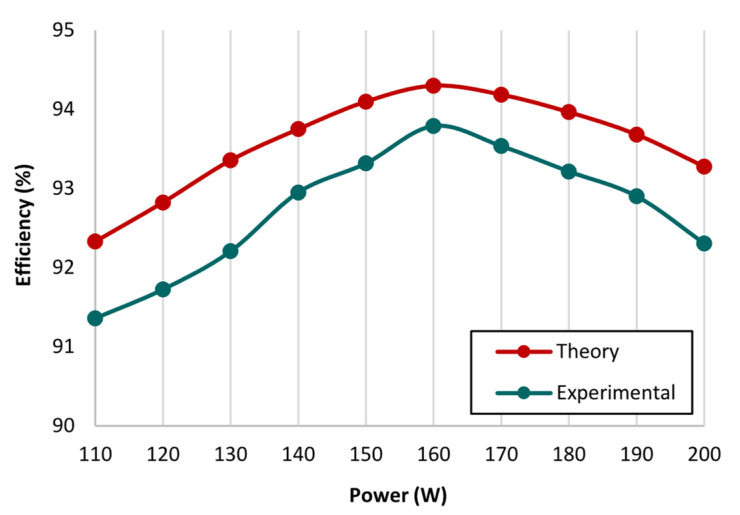
Output power versus efficiency comparison related to theoretical and experimental results.

To verify and evaluate the theoretical results of the proposed converter topology, a 160 W prototype has been developed, as depicted in Figure 8. The design parameters and component specifications are illustrated in Table 2. To operate the proposed converter topology with appropriate switching gate pulses, phase delay, and duty ratio, an Arduino UNO is integrated in the hardware setup. The gate pulses *V_GS_*_X_, *V_GS_*_Y_, and *V_GS_*_Z_ to regulate the switches *S*_X_, *S*_Y_, and *S*_Z_ are depicted in Figure 9a. The two switches *S*_X_ and *S*_Y_ are operated by gate pulses *V_GS_*_X_ and *V_GS_*_Y_ using a switching frequency of 50 kHz with a duty ratio *D*_1_ of 0.5. The switch *S*_Z_ is operated by gate pulse *V_GS_*_Z_ with a 180° phase shift, a similar switching frequency of 50 kHz, and a duty ratio *D*_2_ of 0.35. The input voltage and input current results *V_in_* and *I_in,_* along with the gate pulses are shown in Figure 9b. The proposed converter topology achieved a voltage gain of 19.7 and a voltage ripple of 2.2% while the input voltage is 20 V. During the converter operation, the average input is observed to be 10 A. Furthermore, the theoretical calculation of voltage gain is substantiated by the experimental setup. Figure 9c depicts the output response of the voltage and output current *V_o_* and *i_o_*, along with the gate pulses. The observed average output current is 400 mA. Additionally, the current ripple for both the inductors *L*_X_ and *L*_Y_ is observed to be 8%. Figure 9d illustrates the voltage stress across the *S*_X_ and *S*_Y_ along with the gate pulse; it is evident that the switching stress is 25% related the output voltage. Whereas, for the switch *S*_Z_, switching stress is half or 50% related the output voltage. Figure 9e depicts the response of inductor currents *I_LX_* and *I_LY_* along with the gate pulses; the inductor currents are observed to be continuous. For the ON-state duration of the two switches *S*_X_ and *S*_Y_, the source current is almost double the inductor current *I_LX_* or *I_LY_*, and for the OFF-state duration of the two switches *S*_X_ and *S*_Y_, the inductor current *I_LX_* or *I_LY_* is similar to the input current. Considering the topologies presented in Table 1, the proposed converter topology persists with a significantly low switching stress percentage in comparison to the output voltage. Diode stress *V_D_*_1_ and *V_D_*_2_ are shown in Figure 9f. For the diode *D*_1_, the maximum voltage stress is similar to the input voltage; for diode *D*_2_ the maximum voltage stress is equivalent to the average source voltage and the output voltage. Figure 10 depicts the output power and efficiency comparison. The analysis is based on the theoretical and experimental setup; the observed deviation is about 1.08%, between the theoretical and experimental investigation. The hardware prototype results validate the efficacy of the proposed converter; it is capable of generating high voltage gain with minimum voltage stress on the diodes and the switches.

## 7. Conclusions

A novel non-isolated high-gain non-inverting interleaved converter topology is proposed in this paper. DC–DC converters utilizing a single duty ratio *D* persist with implications while operating at excessive duty cycle ratios. Considering this issue, the proposed converter includes three switches and these switches are operated with two distinct duty ratios, avoiding excessive duty cycle ratios. The proposed converter topology also has some limitations that include: a slightly higher component count, and it is not recommended for applications that require lower voltage gain or lower loads that operate the converter in discontinuous conduction mode (DCM). However, in contrast, the proposed converter topology is designed to generate a higher voltage gain with a nominal voltage stress, and it is suitable for high power applications and to operate in continuous conduction mode (CCM). Theoretical and practical analyses for the proposed converter topology were carried out considering the crucial parameters, such as voltage stress, on the diodes and switches, voltage gain, and efficiency. Thus, to validate the performance analysis practically, a prototype model 160 W 20/400 V of the proposed converter was built for experimental setup. The voltage gain percentage for the proposed converter is significantly higher with lower voltage stress on the switches and diodes with respect to the recently developed DC–DC converter topologies. The maximum efficiency of the proposed converter is well above 90%. Therefore, the pivotal features of the proposed converter determine it to be an appropriate choice for voltage boosting applications, such as renewable energy applications, energy management of the microgrid, fuel cell-based energy generation, and electric automobiles. Further improvements can be made in the proposed converter topology by designing it for low power applications by changing the positions of the switches and their operation, provided that if offers low voltage stress on the components, as compared to established converter topologies.

## Figures and Tables

**Table 1:** Steady-state analysis and the component count comparison.

Converter Topologies	Voltage Gain	Voltage Gain (%)	Switching Stress	Diode Stress	Switches	Inductors	Diodes	Capacitors
Conventional Boost Converter [8]	$\frac{1}{\left(1-D\right)}$	10	$V_{DS}=V_{out}$	$V_{0}$	1	1	1	1
Converter in ref. [22]	$\frac{1+D}{{\left(1-D\right)}^{2}}$	12	$V_{DS}=V_{out}$	$\begin{array}{c} V_{D1}=V_{C1}\\ V_{D2}=\frac{V_{out}+V_{C1}}{1+n_{2}}\\ V_{D3}=V_{out}+nV_{C1}\\ V_{D4}=V_{C2}\;\\ V_{D5}=V_{out}-V_{C2}\\ V_{D6}=V_{out}-V_{C3} \end{array}$	1	4	6	4
Converter in ref. [23]	$\frac{\left(3D_{1}-2D_{2}\right)}{\left(1-D_{1}-D_{2}\right)}$	11.11	$\begin{array}{c} V_{DSX}=V_{DSY}=\frac{V_{out}-V_{in}}{2}\\ V_{DSZ}=V_{out}-2V_{in} \end{array}$	$V_{D1}=V_{2}=-(V_{out}-V_{in}$)$V_{D3}=\frac{-\left(V_{out}-V_{in}\right)}{2}$	3	2	3	3
Converter in ref. [30]	$\frac{1+D}{1-D}$	3	$\begin{array}{c} V_{DSX}=V_{DSY}=\frac{V_{out}+V_{in}}{2}\\ V_{DSZ}=V_{out}+V_{in} \end{array}$	-	3	2	0	1
Converter in ref. [31]	$\frac{\left(1+D_{1}\right)}{\left(1-D_{1}-D_{2}\right)}$	10	$V_{DSX}=V_{DSY}=\frac{V_{out}+V_{in}}{2}$ $V_{DSZ}=V_{out}$	$V_{D1}=V_{in}$ $V_{D2}=V_{in}+V_{out}$	3	2	2	2
Converter in ref. [32]	$\frac{\left(2-D_{1}\right)}{\left(1-D_{1}-D_{2}\right)}$	10.52	$V_{DSX}=V_{DSY}=\frac{V_{out}}{2}$ $V_{DSZ}=V_{out}$	$V_{D1}=V_{D2}=\frac{V_{out}}{2}$ $V_{D3}=V_{out}$	3	2	3	2
Converter in ref. [33]	$\frac{2}{\left(1-D_{1}-D_{2}\right)}$	13.33	$V_{DSX}=V_{DSY}=\frac{V_{out}}{2}$	$V_{D1}=V_{D2}=V_{D3}=V_{D4}=\frac{V_{out}}{2}$	2	2	4	4
Converter in ref. [34]	$\frac{\left(1+D\right)}{\left(1-D\right)}$	8.3	$V_{DSX}=V_{DSY}=\frac{V_{in}+V_{out}}{2}$	$V_{D}=V_{in}+V_{out}$	2	2	1	1
Proposed Converter	$\frac{2\left(1+D_{1}\right)}{\left(1-D_{1}-D_{2}\right)}$	20	$\begin{array}{c} V_{DSX}=V_{DSY}=\frac{V_{in}+V_{out}}{4}\\ V_{DS3}=\frac{V_{out}}{2}\; \end{array}$	$V_{D1}=V_{in}$ $V_{D2}=V_{D3}=V_{D4}=\frac{V_{in}+V_{out}}{2}$	3	2	4	3

**Table 2:** Proposed converter’s design parameter.

Parameters	Ratings (Units)
Rated Power	160 W
Source Voltage	20 V
Output Voltage	400 V
Duty Cycle Ratio (*D*_1_)	50%
Duty Cycle Ratio (*D*_2_)	35%
Switching Frequency (*f_sw_*)	50 kHz
Inductors (*L*_X_, *L*_Y_)	360 μH
Capacitor (*C*_1_, *C*_2_, *C*_o_)	100 μF
Switches (*S*_X_, *S*_Y_, *S*_Z_)	600 V MOSFET FCP20N60
Diodes (*D*_1_, *D*_2_, *D*_3_, *D*_4_)	10A10 Power Diodes

## Data Availability

Not applicable.

## Nomenclature

*V_in_*	Input voltage	*r_D_*_2_	Parasitic resistance of diode *D*_2_
*V_out_*	Output voltage	*r_D_*_3_	Parasitic resistance of diode *D*_3_
*I_in_*	Input current	*r_D_*_4_	Parasitic resistance of diode *D*_4_
*I_cout_*	Output capacitor current	*V_LX_*	Inductor voltage *L*_X_
*L*_X_	Inductor *L*_X_	*V_LY_*	Inductor voltage *L*_Y_
*L*_Y_	Inductor *L*_Y_	*I_LX_*	Inductor current *L*_X_
*C*_1_	Capacitor *C*_1_	*I_LY_*	Inductor current *L*_Y_
*C*_2_	Capacitor *C*_2_	*V_GS_*_X_	Switching voltage for switch *S*_X_
*C*_o_	Output Capacitor *C*_o_	*V*_GSY_	Switching voltage for switch *S_Y_*
*S*_X_	Switch *S*_X_	*V_GS_*_Z_	Switching voltage for switch *S*_Z_
*S*_Y_	Switch *S*_Y_	*V_DS_*_X_	Voltage stress at switch *S*_X_
*S*_Z_	Switch *S*_Z_	*V_DS_*_Y_	Voltage stress at switch *S*_Y_
*D*_1_	Diode 1	*V_DS_*_Z_	Voltage stress at switch *S*_Z_
*D*_2_	Diode 2	*V_D_*_1_	Voltage stress at diode *D*_1_
*D*_3_	Diode 3	*V_D_*_2_	Voltage stress at diode *D*_2_
*D*_4_	Diode 4	*V_D_*_3_	Voltage stress at diode *D*_3_
*R_o_*	Load resistance	*V_D_*_4_	Voltage stress at diode *D*_4_
*f_sw_*	Switching frequency	*I_C_*_1_	Capacitor current *C*_1_
*r_LX_*	Parasitic resistance of inductor *L*_X_	*I_C_*_2_	Capacitor current *C*_2_
*r_LY_*	Parasitic resistance of inductor *L*_Y_	*P_in_*	Input power
*r_SX_*	Parasitic resistance of switch *S*_X_	*P_out_*	Output power
*r_SY_*	Parasitic resistance of switch *S*_Y_	*P_sw_*	Switching power losses
*r_SZ_*	Parasitic resistance of switch *S*_Z_	*P_rc_*	Capacitor power losses
*r_D_*_1_	Parasitic resistance of diode *D*_1_	*η*	Efficiency
